# Patterns of Mitochondrial ATP Predict Tissue Folding

**DOI:** 10.1101/2025.08.31.673364

**Published:** 2025-08-31

**Authors:** Bezia Lemma, Megan Rothstein, Pengfei Zhang, Bridget Waas, Marcus Kilwein, Safiya Topiwala, Sherry X. Zhang, Anvitha Sudhakar, Katharine Goodwin, Elizabeth R. Gavis, Ricardo Mallarino, Andrej Kosmrlj, Celeste M. Nelson

**Affiliations:** 1 Departments of Chemical & Biological Engineering, Princeton University, Princeton, NJ 08544; 2 Department of Molecular Biology, Princeton University, Princeton, NJ 08544; 3 Department of Omenn-Darling Bioengineering Institute, Princeton University, Princeton, NJ 08544; 4 Departments of Mechanical & Aerospace Engineering, Princeton University, Princeton, NJ 08544; 5 Department of Lewis-Sigler Institute for Integrative Genomics, Princeton University, Princeton, NJ 08544

**Keywords:** Energy metabolism, glycolysis, mechanical stress, reaction-diffusion modeling, tissue morphodynamics

## Abstract

The construction of complex tissue shapes during embryonic development results from spatial patterns of gene expression and mechanical forces fueled by chemical energy from ATP hydrolysis. We find that chemical energy is similarly patterned during morphogenesis. Specifically, mitochondria are locally enriched at the apical sides of epithelial cells during apical constriction, which is widely used across the animal kingdom to fold epithelial tissues. Timelapse imaging, spatial transcriptomics, and measurements of oxygen consumption rate reveal that mitochondrial density, potential, and ATP increase in epithelial cells prior to actomyosin contraction and tissue folding, which is prevented by inhibiting oxidative phosphorylation. Mitochondrial enrichment and apicobasal patterning are conserved during apical constriction in flies, chicks, and mice, and these subcellular patterns can be used to predict computationally patterns of tissue folding. These findings highlight a spatial dimension of bioenergetics in embryonic development.

Morphogenesis is a physical process in which tissues change shape in response to spatiotemporal patterns of gene expression and mechanical force (1, 2). While it is axiomatic that these processes require chemical energy in the form of ATP, surprisingly little is known about the spatiotemporal regulation of energy production, particularly by mitochondria (3). Recent attention has focused on the appearance of glycolytic gradients in chicken and mouse embryos, including during delamination of neural crest cells (4), migration of epiblast cells into the primitive streak (5), and posterior elongation of the body axis (6–8). These gradients have been interpreted in the context of metabolic signaling, as through the Wnt pathway (9), rather than in the context of energetics, although it has been postulated that glycolytic gradients may provide ATP for actin polymerization and thus promote cell motility (6, 10). The direct role of energy metabolism in powering the fundamental morphogenetic processes that fold tissue shapes, especially through mitochondrial production of ATP, remains largely unexplored.

One such fundamental morphogenetic process is apical constriction, a conserved motif in which epithelial tissues fold when cells shrink their apical surfaces by actomyosin contraction (11–13). The molecular machinery that generates the forces necessary for apical constriction is also conserved: ATP hydrolysis leads to phosphorylation of the myosin regulatory light chain and promotes assembly and activity of myosin motors, which contract the actin network that is anchored by adherens junctions to the apical cortex of the cell. During this morphogenetic process, myosin phosphorylation and the generation of force are spatially patterned both within the cell (at the apical side) and within the tissue (in the actively contracting cells). That myosin motors require ATP hydrolysis prompts the question of whether mitochondrial energy metabolism is similarly spatially patterned.

To investigate the possible spatial relationship between mitochondria and generation of mechanical forces during apical constriction, we began by using the embryonic chicken lung as a model system. The early embryonic chicken lung consists of tubes of simple columnar epithelium surrounded by mesenchyme (14). The first three branches form at predictable locations and times along the dorsal surface of the primary bronchus (Fig. S1a–c) (15–17). Epithelial cells at these locations undergo apical constriction (Fig. 1a), which folds the epithelium into the adjacent mesenchyme and thus initiates a branch (16). We took advantage of this stereotypy to systematically visualize mitochondrial membrane density within the epithelium during morphogenesis. Specifically, we isolated embryonic lungs at timepoints in between initiation of the first branch (t=116 hr; Hamburger-Hamilton stage HH24) and initiation of the third branch (t=128 hr; HH27). As a metric for mitochondrial membrane density, we conducted immunofluorescence analysis for Tom20, an outer mitochondrial membrane import receptor subunit (Fig. 1b). This immunostaining revealed that mitochondrial membrane density is enriched in the epithelium within nascent branches. Counterstaining each sample for E-cadherin allowed us to generate three-dimensional (3D) segmentations of the epithelial tree to quantify Tom20 enrichment (Fig. 1b, c; **Movie S1**). Quantitative image analysis confirmed that mitochondrial membrane density is significantly higher in the epithelium than in the mesenchyme, and significantly higher in the medial epithelium that forms branches than in the proximal epithelium that does not (Fig. 1d). Consistently, unbiased spatial transcriptomics analysis revealed that mitochondrial gene expression is enriched within the branching epithelium (Fig. S2a–f). The spatial patterning of elevated mitochondrial density within the branching dorsal epithelium persisted when we normalized to the adjacent non-branching ventral epithelium across all stages examined (Fig. 1e–f; Fig. S1d–f). Curiously, we noticed a strong bias in mitochondrial localization within the branching epithelium, with elevated density on the apical side of the cells (Fig. 1g, insets; Fig. S1g–i), mirroring the patterns of actomyosin contraction at the apical surfaces of these regions of the tissue.

To directly visualize mitochondrial energy metabolism, we took advantage of the fact that embryonic chicken lungs continue to undergo branching morphogenesis when cultured as explants (15–18). We isolated and cultured embryonic lungs in the presence of MitoView633, a mitochondrial membrane potential-dependent dye that accumulates at mitochondria and fluoresces in proportion to the voltage across the mitochondrial membrane (19) (Fig. 1h). As with staining for Tom20, we quantified the relative fluorescence intensity of MitoView633 as a function of position along the primary bronchus by normalizing to the adjacent ventral epithelium (Fig. 1i). We found that mitochondrial membrane potential is higher in the dorsal epithelium than in the non-branching ventral epithelium. Consistent with our analysis of mitochondrial membrane density and mitochondrial gene expression, we found that mitochondrial membrane potential is elevated at the apical sides of cells within branch sites in the epithelium, even prior to branch initiation (Fig. 1h; Fig. S1j–m; **Movie S2-S3**). These data are consistent with an increase in mitochondrial activity – and potentially higher levels of chemical energy in the form of ATP – within the epithelium as it undergoes apical constriction.

To determine directly whether sites of apical constriction correspond to elevated levels of ATP, we cultured lung explants in the presence of ATPRed1, which labels ATP surrounding mitochondria (20). We first calibrated the dye by measuring its fluorescence intensity as a function of ATP concentration (Fig. S3a). We also confirmed that blocking ATP synthesis by treating with the mitochondrial ATP synthase inhibitor, oligomycin, leads to a decrease in ATPRed1 fluorescence intensity (Fig. S3b–d). We then cultured embryonic lung explants in the presence of ATPRed1 during the branching process (Fig. 2a; **Movie S4**). We found that the fluorescence intensity of ATPRed1 is lowest in the non-branching epithelium, increases at future branch sites prior to apical constriction, and further increases within the epithelium of nascent branches (Fig. 2b–c; Fig. S3e; **Movie S5**). Blocking actomyosin contractility by treating explants with the myosin II inhibitor, blebbistatin, blocks both apical constriction as well as the formation of new branches without preventing epithelial growth (Fig. 2d; Fig. S3f–g) (16, 21). Curiously, we found that treatment with blebbistatin decreases mitochondrial ATP production, as inferred by quantifying the fluorescence intensity of ATPRed1 (Fig. 2e–f; **Movie S6**), but has no effect on mitochondrial membrane density, as inferred by quantifying Tom20 staining intensity (Fig. 2g). These data suggest that the process of apical constriction both activates and is coupled to mitochondrial ATP synthesis within the actively contracting region of the epithelium, even before detectable deformation of the tissue.

Mitochondrial ATP synthesis relies on a proton gradient across the mitochondrial membrane, maintained by oxygen molecules accepting electrons at the end of the electron transport chain. Our data thus suggested that regions of the epithelium undergoing apical constriction might consume more oxygen than other regions of the tissue. To test this hypothesis, we surgically divided embryonic chicken lung explants into proximal (non-branching), medial (branching), and distal (future branching) regions (Fig. 2h), and then used Seahorse analysis to measure the oxygen consumption rate of each region separately (Fig. S4a–i). This analysis revealed a significantly higher oxygen consumption rate in the medial branching region of the lung, as compared to the proximal non-branching region (Fig. 2i), consistent with our hypothesis. As expected from our experiments using ATPRed1, we found that blocking apical constriction by treating explants with blebbistatin significantly attenuated the oxygen consumption rate of the branching medial region (Fig. 2i). Actomyosin-induced apical constriction thus coincides with and promotes mitochondrial respiration and synthesis of ATP.

Given that the patterns of mitochondrial density (Tom20 and spatial transcriptomics), membrane potential (MitoView633), ATP (ATPRed1), and oxygen consumption rate (Seahorse) either correspond to or presage those of apical constriction in the embryonic chicken lung epithelium, we hypothesized that mitochondrial respiration might be required for this developmental process (Fig. 3a). We tested this hypothesis by culturing embryonic lung explants in the presence of oligomycin, which halved the oxygen consumption rate within 2 hours after administration (Fig. 3b; Fig. S4c), consistent with a significant reduction in mitochondrial respiration. Strikingly, treatment with oligomycin completely blocked the initiation of new branches in the explants (Fig. 3c, d; **Movie S7**). We also found that treatment with oligomycin led to a reduction in the intensity of staining for both F-actin as well as phosphorylated myosin light chain (pMLC) at the apical surface of the epithelium (Fig. 3e, f). Importantly, blocking mitochondrial respiration had no effect on the spatial patterns of mitochondrial density: oligomycin-treated explants still showed elevated levels of Tom20 staining intensity at branch sites in the dorsal epithelium and on the apical sides of actively branching cells (Fig. 3e–g). Mitochondrial production of ATP is therefore required for the generation of new branches by apical constriction of the embryonic chicken lung epithelium.

Apical constriction is a conserved morphogenetic motif that is used to fold simple epithelial sheets and tubes into more complex geometries, including during gastrulation (22), neurulation (23), and eye development (24, 25). To determine whether spatial patterns of mitochondrial density are conserved during apical constriction, we broadened our analysis across tissues and species. Specifically, we examined ventral furrow formation during gastrulation in *Drosophila* (Fig. 4a), neural tube closure during neurulation in chicken (Fig. 4b), and lens placode invagination during eye development in mouse embryos (Fig. 4c). Staining each system for Tom20 revealed spatial patterns of mitochondrial membrane density almost identical to those we observed in the chicken lung epithelium, with elevated intensity in the future ventral furrow (Fig. 4d), neural tube (Fig. 4e), and optic lens (Fig. 4f), and a bias to the apical side of the epithelium (Fig. 4g–j). Notably, mitochondrial accumulation in the ventral furrow aligns with previous observations of mitochondrial repositioning and fusion (26). Apical enrichment of mitochondria thus appears to be a defining feature of epithelial cells undergoing apical constriction.

To determine whether the apicobasal patterning of mitochondrial density can be used to predict subcellular patterns of ATP concentration and, thus, actomyosin kinetics, we constructed a reaction-diffusion model of the cell. Specifically, we assumed that ADP is converted into ATP as a function of local mitochondrial density, at a rate determined by our Seahorse measurements, and hydrolyzed as a function of actomyosin and ATP concentrations, according to Michaelis-Menten kinetics. These mass-action kinetics predict a higher ATP/ADP ratio at the apical side of the cell along with a correspondingly higher rate of myosin phosphorylation (Fig. 4k–l). To test this prediction experimentally, we transduced embryonic chicken lungs with PercevalHR, a genetically encoded fluorescent biosensor that reports the intracellular ATP/ADP ratio (27). We then used this reporter to measure the ATP/ADP ratio during apical constriction and found, indeed, a significantly higher ratio at the apical side (Fig. 4m). To determine whether these subcellular patterns of mitochondria and rates of myosin phosphorylation might be sufficient to promote the observed multicellular patterns of folding, we then used the finite element method (FEM) to construct a 3D continuum model of each developing tissue, based on geometrical and biophysical parameters. Each tissue started as a simple shape (ellipsoid, half cylinder, or sheet, respectively), onto which we mapped patterns of apical constriction, as predicted by our observations of the subcellular patterns of mitochondria (Fig. 4n; Fig. S5a–b). Remarkably, each simulation produced a final geometry similar to that observed experimentally (Fig. 4o; Fig. S5c–d; **Movie S8**), suggesting that apicobasal patterns of mitochondrial density can be used to predict patterns of mechanical forces that drive changes in tissue shape. To determine whether we could similarly predict patterns of chemical energy within each developing system, we conducted timelapse imaging analysis of ATPRed1-labeled explants (Fig. 4p). In each case, we found significantly elevated fluorescence intensity in the regions of tissue predicted to undergo apical constriction (Fig. S5e–j). Collectively, our data suggest that the chemical energy from mitochondrial respiration is biased towards the apical sides of cells and coupled to the forces of tissue folding during apical constriction.

Mitochondrial density, membrane potential, and ATP production are elevated at the apical sides of cells before and during apical constriction. Our reaction-diffusion and FEM models suggest that the apical concentration of mitochondria would be sufficient to promote myosin phosphorylation at the apical cortex of the cell, thus promoting tissue folding. These observations, combined with previous studies focused on the molecular components of the force-generating machinery, suggest that the morphogenetic motif of apical constriction involves a conserved network in which spatially patterned energy metabolism is dynamically integrated with conserved genetic and mechanical components fold epithelia (28, 29). Importantly, we observe that the apicobasal patterning of mitochondrial density can be used to predict which cells within the epithelium will undergo apical constriction before appreciable changes in cell or tissue shape. Our findings suggest a new general paradigm: that subcellular patterns of organelles, energy, and energy metabolism are upstream of changes in tissue morphology during development. Consistent with this notion, waves of glucose uptake and glycolytic metabolism have been found to precede induction of epithelial-mesenchymal transition (EMT), a different morphogenetic motif that is important for development of the neural crest, primitive streak, and presomitic mesoderm (4–10), possibly downstream of signaling through fibroblast growth factor (FGF) (6, 30, 31). The upstream signals that promote the apical bias in mitochondrial localization prior to apical constriction remain to be uncovered.

Mitochondrial ATP production also appears to be essential for apical constriction, as treatment with oligomycin blocks actomyosin contraction. This observation could be taken to suggest that mitochondrial respiration is enhanced in these cells in lieu of glycolysis, as has been proposed for differentiating cells in the developing retina (32). However, our spatial transcriptomics and Seahorse data suggest that branching epithelia express high levels of glycolytic enzymes (Fig. S2g) and show elevated glycolytic extracellular acidification rates (Fig. S4f, S4m–o), suggesting a possible coupling between glucose metabolism and mitochondrial respiration, at least in the developing lung. While mitochondrial ATP is clearly necessary, quantifying the relationship between energy supply and mechanical work raises questions about efficiency. Estimations of the viscoelastic energy requirements necessary for apical constriction to deform the airway epithelium into a branch (**Methods** and **Movie S8**) suggest that the energy provided by mitochondrial respiration and glycolysis is staggeringly large (~100,000,000 nJ) compared to the morphological deformation itself (~10 nJ; **Methods**). A more accurate estimate will require teasing apart the energetic budget of the tissue and accounting for its energy demands beyond actomyosin contraction at the apical cortex. Nonetheless, this difference is vast, and might be explained by the low efficiencies with which energy is transduced into systematic molecular motion and force (33, 34). Alternatively, glycolysis and/or oxidative metabolism might promote biochemical signaling downstream of morphogens or reactive oxygen species to initiate apical constriction (4, 35). It will be intriguing to uncover both the upstream and downstream signals that link chemical energy, mechanical forces, and tissue folding.

## Supplementary Material

1

## Figures and Tables

**Fig. 1.**
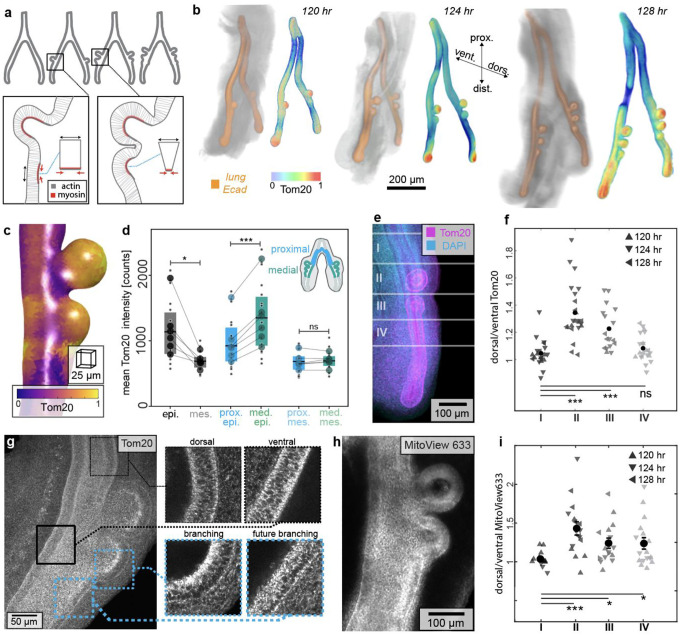
Mitochondrial membrane density and potential are locally enriched in the lung epithelium at branch sites prior to and during apical constriction. (**a**) Schematic of early embryonic chicken lung, indicating sites of apical constriction. (**b**) Projections of confocal images of staining for E-cadherin (orange, left) and Tom20 (color-coded, right) at different stages of development. Scale bar, 200 μm. (**c**) Local intensity of Tom20 within the epithelium, color-coded based on intensity. Scale bar, 25 μm. (**d**) Graph of Tom20 staining intensity in the entire epithelium and mesenchyme, proximal (non-branching) epithelium, medial (branching) epithelium, and adjacent mesenchymal regions. Shown are data from 18 lungs (small circles) across 6 independent replicates (means shown by large circles). Lines match independent replicates between measurements. Box plot shows median, first, and third quartile of data. *, *** indicate p=0.02, p=0.001 respectively. (**e**) Maximum-projection image of staining for Tom20 (magenta) and nuclei (blue), partitioned into four regions: (I) non-branching, (II) branching, (III) nascent branching, and (IV) future branching. Scale bar, 100 μm. (**f**) Graph of normalized Tom20 staining intensity in regions (I-IV) of lungs at different stages of development. Shown are data from 21 lungs across 9 independent replicates. *** indicates p=2e-7, p=9e-5 respectively (**g**) Maximum-intensity projection of staining for Tom20; insets show intensity and apicobasal patterning in single z-planes. Scale bar, 50 μm. (**h**) Maximum-intensity projection of MitoView633 fluorescence. Scale bar, 100 μm. (**i**) Graph of normalized MitoView633 intensity in regions (I-IV) of lungs at different stages of development. Shown are data from 17 lungs. ***, *, * indicate p=0.0003, p=0.01, and p=0.03 respectively.

**Fig. 2.**
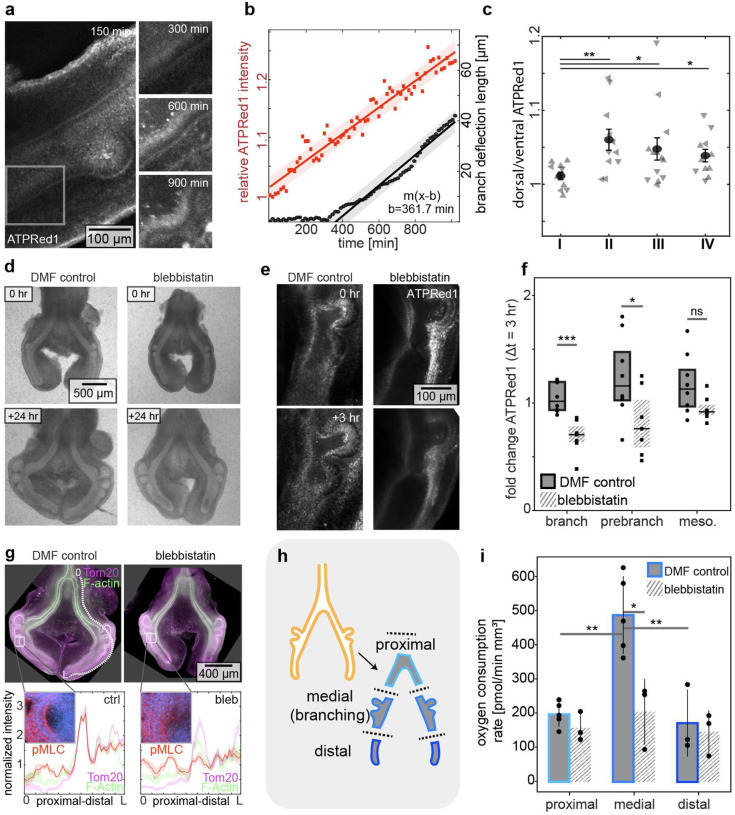
Mitochondrial ATP density and oxygen consumption rate are increased at sites of branch initiation both before apical constriction and in response to actomyosin contraction. (**a**) Timelapse images of ATPRed1 intensity during branch initiation in the embryonic chicken lung. Scale bar, 100 μm. (**b**) Graph showing ATPRed1 intensity at the dorsal side relative to the ventral side (red) and deflection of the epithelium (black) as a function of time, for the inset region shown in (a). Shaded band represents 95% confidence interval of linear fit. (**c**) Graph of normalized ATPRed1 intensity in regions (I-IV; defined in Fig. 1e) of lungs at different stages of development. Shown are data from 17 lungs. **, *, * indicate p=0.001, p=0.04, and p=0.02, respectively. (**d**) Phase-contrast images of lung explants before and 24 hours after culture in the presence of DMF control or blebbistatin (20 μM). Scale bar, 500 μm. (**e**) Maximum intensity projections of ATPRed1 staining immediately after and 3 hours after culture in the presence of DMF control or blebbistatin (20 μM). Scale bar, 100 μm. (**f**) Fold change in ATPRed1 over 2 hours of treatment in dorsal branching tissue. Shown are data from 8 independent control lungs and 7 independent blebbistatin-treated lungs. *, ** indicates p=0.03, and p=0.0004 respectively. (**g**) Top: maximum-intensity projection images of staining for Tom20 (magenta) and F-actin (green) in lungs cultured in the presence of DMF control or blebbistatin. Scale bar, 400 μm. Bottom: insets show pMLC (red) and nuclei (blue) at the region of an initiating branch. Graphs show pMLC from proximal (0) to distal (L) along the outer edge of the epithelium, as indicated by the dotted white line. Data are normalized and averaged over four samples. Shaded region represents standard error of the mean (s.e.m.). (**h**) Schematic of dissections used to divide lungs into proximal (non-branching), medial (branching), and distal (future branching) regions. (**i**) Graph showing oxygen consumption rate of proximal, medial, and distal regions of lungs cultured in the presence of DMF control or blebbistatin. Each dot indicates a replicate, with each replicate containing tissue pooled from 20 lungs. **, *, **, indicate p=0.003, p=0.013 and p=0.002, respectively.

**Fig. 3.**
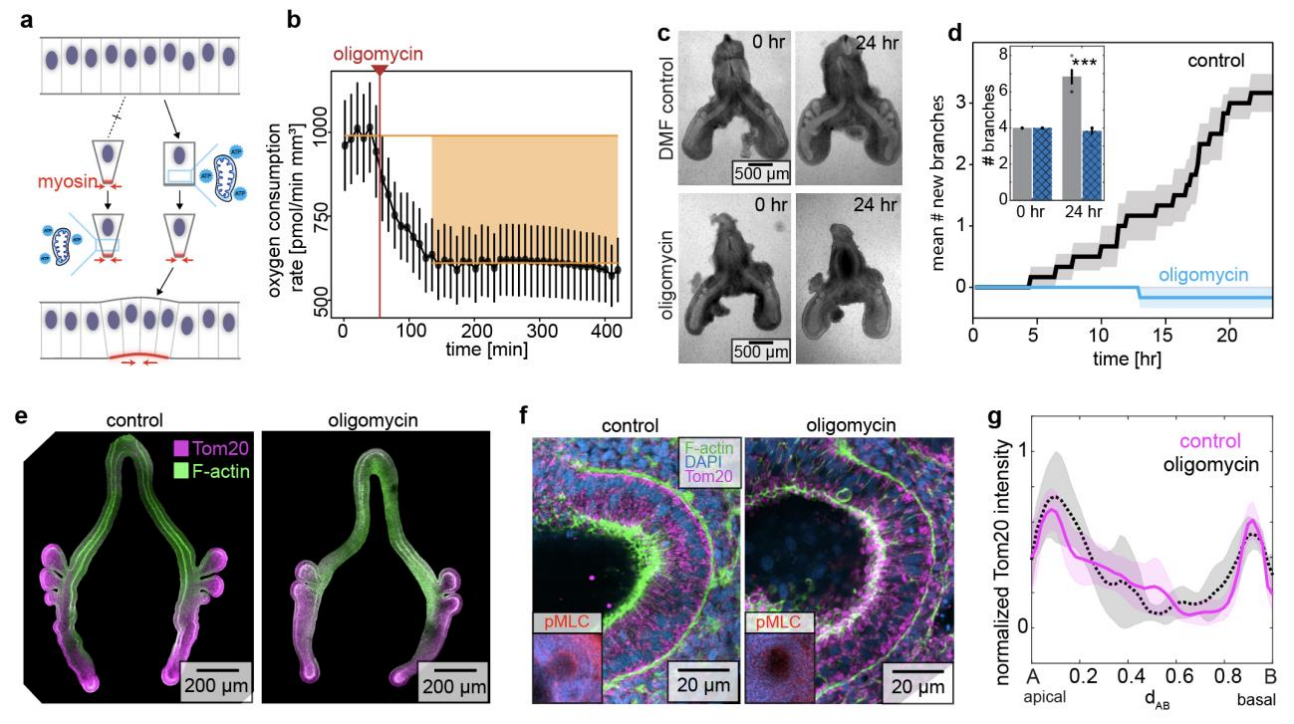
Mitochondrial ATP production is required for apical constriction and branch initiation in the embryonic chicken lung. (**a**) Schematic of two possible connections between mitochondrial energy and apical constriction. Actomyosin-driven apical constriction either precedes (left) or follows (right) mitochondrial ATP production. (**b**) Graph showing oxygen consumption rate of lungs as a function of time before and after treatment with oligomycin (2 μM). (**c**) Phase-contrast images of lung explants before and 24 hours after culture in the presence of DMF control or oligomycin (2 μM). Scale bars, 500 μm. (**d**) Graph showing the mean number of new branches as a function of time in lung explants cultured in the presence of DMF control or oligomycin (2 μM), averaged over 6 independent experiments. Shaded error bar indicates s.e.m. Inset bar graph shows initial and final number of branches. *** indicates p=0.000016. (**e**) Fluorescence images of staining for Tom20 (magenta) and F-actin (green) in lung explants cultured for 12 hours in the presence of DMF control or oligomycin (2 μM). Scale bars, 200 μm. (**f**) Fluorescence images of staining for Tom20 (magenta), F-actin (green), and DAPI (blue). Insets show: DAPI (blue) and pMLC (red). Scale bars, 20 μm. (**g**) Graph showing quantification of normalized Tom20 intensity as a function of position along the apicobasal axis of the branching epithelium, manually aligned for 3 independent staining experiments. Shaded error bar indicates s.e.m. at each position.

**Fig. 4.**
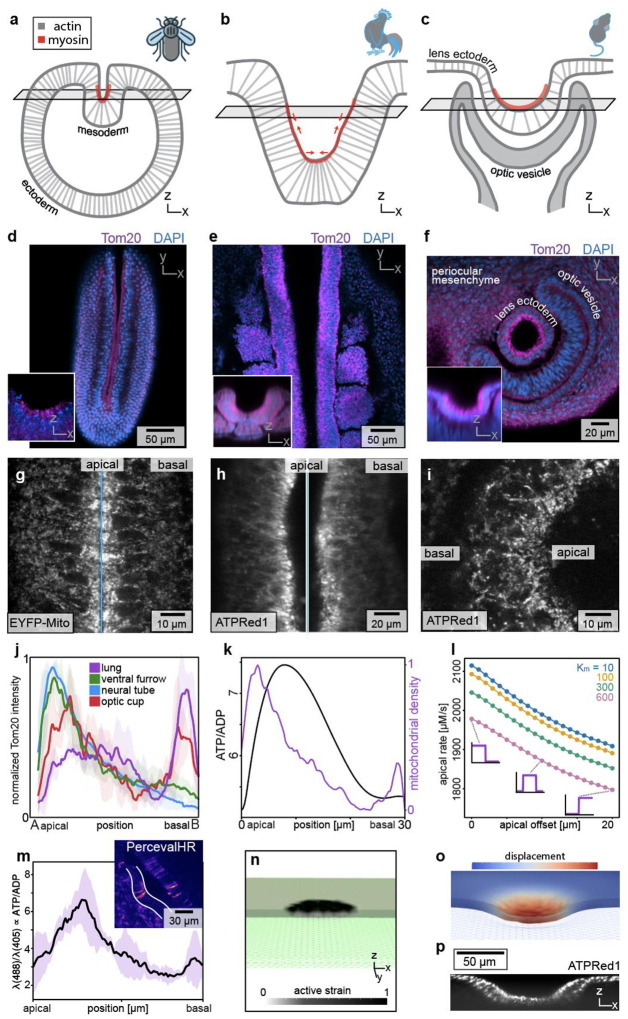
Patterns of mitochondrial membrane density are conserved and predict apical constriction across species and tissues. Schematics of (**a**) ventral furrow formation during gastrulation in *Drosophila*, (**b**) closure of the neural tube during neurulation in chicken, and (**c**) invagination of the lens placode during eye development in mouse embryos. (**d-f**) Confocal images showing staining for Tom20 (magenta) and nuclei (blue) during gastrulation, neurulation, and lens invagination. Insets show z-x sections. Scale bars 50 *μ*m, 50 *μ*m, and 20 *μ*m. (**g-i**) Confocal images showing live imaging of apicobasal mitochondrial patterning, as reported by EYFP-Mito in Drosophila, and ATPRed1 in chicken neural tube and mouse optic cup. Scale bars 10 *μ*m, 20 *μ*m, and 10 *μ*m. (**j**) Graph showing normalized Tom20 intensity in each tissue, revealing enrichment of mitochondria on the apical side. (**k**) Graph showing the ATP/ADP ratio (black) resulting from a mitochondrial density pattern (purple) in a 1D reaction-diffusion model, with Michaelis-Menten kinetics of hydrolysis on the apical side due to actomyosin in the region indicated in red and a baseline rate of hydrolysis throughout the cell. (**l**) Graph showing that the reaction-diffusion model predicts a decrease in myosin phosphorylation rate as the mitochondrial distribution is moved from the apical to the basal side of the cell, plotted for different values of the Michaelis constant *K*_*m*_. (**m**) Graph showing measurements of ATP/ADP ratio in cells undergoing apical constriction in branching epithelium of the chicken lung. Inset: confocal ratiometric image of PercevalHR. Scale bar 30 *μ*m. (**n**) Cutaway view of initial geometry for a simulation based on mitochondrial patterning of tissue deformation in the mouse optic cup; numerical mesh drawn in green, with applied active stress shown in grayscale. (**o**) Cutaway view of mesh deformed by active stresses, colored by the displacement field. (**p**) Confocal z-slice reconstruction of ATPRed1 signal in the mouse optic cup. Scale bar 50 *μ*m.

## Data Availability

All data needed to evaluate the conclusions in the paper are present in the main text and/or the Supplementary Materials.

## Abbreviations:

3D: three-dimensional
FEM: finite element method
HH: Hamburger-Hamilton
pMLC: phosphorylated myosin light chain
