# Supplementary material for: Patterns of Mitochondrial ATP Predict Tissue Folding
Source: bioRxiv. 2025 Aug 31:2025.08.31.673364. Preprint. [Version 1] doi: 10.1101/2025.08.31.673364 (PMC12407887; doi:10.1101/2025.08.31.673364)
Supplement: 1 [file NIHPP2025.08.31.673364V1-supplement-1.pdf]

bioRxiv preprint doi: https://doi.org/10.1101/2025.08.31.673364; this version posted August 31, 2025. The copyright holder for this preprint (which was not certified by peer review) is the author/funder, who has granted bioRxiv a license to display the preprint in perpetuity. It is made available under aCC-BY 4.0 International license.

Supplementary Materials

Materials and Methods

Animal husbandry

All procedures involving animals complied with ethical regulations and were approved by the Princeton University Institutional Animal Care and Use Committee (IACUC). For experiments with embryonic lungs and neural tubes, fertilized chicken (*Gallus gallus* variant *domesticus*) eggs were obtained from the Department of Animal Science, University of Connecticut. Eggs were incubated in an upright configuration at 37°C and 70% humidity for the specified number of hours. Underdeveloped or deformed embryos were discarded. For experiments with embryonic eyes, C57BL6/J mice were bred and maintained under standard laboratory conditions, receiving food and water *ad libitum* in an AAALAC-accredited facility in accordance with the NIH Guide for the Care and Use of Laboratory Animals. Noon of the day on which a vaginal plug was detected was considered as embryonic day 0.5 (*E*0.5). For experiments with gastrulating embryos, *Drosophila melanogaster* strains were reared at 21°C under standard laboratory conditions. For embryo collection, flies were transferred into cages and fed using agarose plates supplemented with apple juice and yeast paste.

Wholemount immunofluorescence staining and imaging

Embryonic tissues were isolated and fixed in 4% paraformaldehyde (PFA) with 0.25% glutaraldehyde in phosphate-buffered saline (PBS) with at least a 1:20 ratio of sample volume to fixative volume for 30 min at 4°C on a shaker, followed by an additional 15 min at 20°C on a shaker. Samples were washed in tris-buffered saline (TBS) containing 7.35 mg/mL $CaCl_2$ for 15 min on a shaker and then in TBS containing 0.5% Triton X-100 (TBST) three times for 15 min each. Samples were then blocked in blocking buffer comprised of TBST containing 10% donkey serum for 1 hour at 20°C on a shaker. After blocking, samples were incubated in blocking buffer containing primary antibodies against Tom20 (1:100; D8T4N, rabbit mAb; Cell Signaling 42406S), E-cadherin (1:500; clone 36, mouse mAb; BD 610182), or pMLC (1:50; Ser19, mouse mAb; Cell Signaling 3675S) for 48 hours at 4°C. Samples were then washed in TBST four times for 15 min each. Samples were then incubated in blocking buffer containing donkey anti-rabbit (H+L) Alexa Fluor 555 (1:500; ThermoFisher Scientific A31572), donkey anti-mouse (H+L) Alexa Fluor 680 (1:500; ThermoFisher Scientific A10038), or Alexa Fluor 488-conjugated

bioRxiv preprint doi: https://doi.org/10.1101/2025.08.31.673364; this version posted August 31, 2025. The copyright holder for this preprint (which was not certified by peer review) is the author/funder, who has granted bioRxiv a license to display the preprint in perpetuity. It is made available under aCC-BY 4.0 International license.

phalloidin (1:500; ThermoFisher Scientific A12379), and then counterstained with DAPI (1:1000; ThermoFisher Scientific D1306). Samples were washed in TBST three times for 15 min each, and then once in PBS for 15 min. Samples were dehydrated in an isopropanol series at 25%, 50%, 75%, and 100% for 15 min each and then titrated into a 1:2 ratio of benzyl alcohol to benzyl benzoate (BA:BB) at 50% and then 100% for 15 min each. Samples cleared in BA:BB were held by a nylon washer attached to two coverslips and imaged using a Hamamatsu MAICO scanning confocal fitted to an inverted Nikon TE microscope with a Ludl stage using a 10× 0.5 *NA* Nikon objective (CFI Super Fluor 10× MRF00101) for whole-organ imaging or a 40× 1.15 *NA* Nikon objective (CFI Apo LWD Lambda 40XC WI MRD77410) for high-magnification imaging.

Explant culture and live-imaging analysis

Embryonic lungs were dissected in sterile PBS and transferred to custom-made imaging chambers consisting of a #1.5 coverslip coated with a thin layer of polyacrylamide (**Fig. S6**). Samples were cultured under a thin layer of DMEM/F12 medium (without HEPES or Phenol Red) supplemented with 5% fetal bovine serum (FBS, heat-inactivated; Atlanta Biologicals) and antibiotics (50 units/mL of penicillin and streptomycin). A 1-mL reservoir of culture media was created by surrounding the explants in a ring of hydrated, sterilized, fibrous material. Timelapse imaging was performed within an OKO stage-top incubator at 37°C, 5% CO_2, and 75% relative humidity. Confocal fluorescence images were acquired using a Hamamatsu MAICO scanning confocal fitted to an inverted Nikon TiE microscope with a Ludl stage and a 20× 0.95 *NA* WI Nikon objective (CFI Apo LWD Lambda S 20XC WI MRD77200). Phase-contrast images were acquired using a 4× 0.2 *NA* objective (CFI Plan Apochromat Lambda D 4X MRD70040); a green filter was placed on the brightfield arm at the condenser to reduce damage from light.

Stock concentrations of MitoView633 (200 mM; Biotium #70055) and ATPRed1 (4.5 mM; Sigma-Aldrich SCT045) were prepared in DMF. To prevent the precipitation of ATPRed1, we generated a final stock of 1.4-mM ATPRed1 by mixing 4 μL of PEG300 (ThermoFisher Scientific 192220010) with 2 μL of prepared dye, followed by adding 0.5 μL of Tween20. Stock mixtures were sonicated for 2 min after preparation and again for 2 min immediately before use.

bioRxiv preprint doi: https://doi.org/10.1101/2025.08.31.673364; this version posted August 31, 2025. The copyright holder for this preprint (which was not certified by peer review) is the author/funder, who has granted bioRxiv a license to display the preprint in perpetuity. It is made available under aCC-BY 4.0 International license.

We used pulled-glass needles (<5 μm in diameter) to create holes in the distal tip of the embryonic lung epithelium. We then loaded 40-μM MitoView633 or 1.1-mM ATPRed1 into pulled-glass needles and injected ~0.1 μL into the carina of the lung for live-imaging analysis (**Fig. S6**). Fluorescence images were acquired 30 min after injection.

For experiments with gastrulating embryos, *Drosophila melanogaster* embryos were collected at the cellularization stage, dechorionated, mounted on a coverslip using heptane glue, and covered with Halocarbon Oil 200 before imaging.

For live-imaging analysis of the neural tube, fertile eggs were incubated for 32 hours such that embryos developed to stage HH8-9. Embryos were collected on filter paper using the EC protocol (*36*) and treated with ATPRed1 and MitoView633 diluted in media (1:1 for ATPRed1; 1:8 for MitoView633), added to the embryonic ectoderm using a 20-μL pipette, locally mixing the dye along the neural tube. Embryos on filter paper were then cultured dorsal side down in a glass-bottom dish in egg albumin at 37°C for live-imaging experiments.

For live-imaging analysis of embryonic mouse eye placodes, embryos were collected at *E*10.5 and placed into glass-bottom plates containing media [DMEM with sodium pyruvate (100 mM), HEPES (1 M), MEM non-essential amino acids, penicillin-streptomycin, and 10% FBS]. ATPRed1 and MitoView633 were first diluted in media (1:1 for ATPRed1; 1:8 for Mitoview633). The embryo was placed on its side, then 10 μl of the diluted dye mixture was pipetted onto the eye placode. The embryo was then flipped so that the eye placode labeled with ATPRed1 and MitoView633 would be in contact with the glass-bottom plate for live-imaging analysis.

To inhibit actomyosin contraction or mitochondrial ATP generation, lung explants were cultured in media containing 1:1000 para-amino-blebbistatin (Cayman #22699) or oligomycin (Sigma-Aldrich #75351) dissolved in DMF at the concentrations indicated. As a vehicle control, lung explants were cultured in media containing 1:1000 DMF.

Virus production and injection

bioRxiv preprint doi: https://doi.org/10.1101/2025.08.31.673364; this version posted August 31, 2025. The copyright holder for this preprint (which was not certified by peer review) is the author/funder, who has granted bioRxiv a license to display the preprint in perpetuity. It is made available under aCC-BY 4.0 International license.

The PercevalHR RCAS expression construct was assembled by subcloning the PercevalHR insert from GW1-PercevalHR (Addgene #49082) into an RACS(BP) avian retroviral backbone. The recombinant RCAS plasmid was transfected into chick DF1 cells in T-75 flasks using standard transfection protocol. The transfected cells were further maintained in T-175 flasks until confluency. Cell culture medium was harvested from confluent populations once per day for four days, and concentrated at 45,000 rpm for 1.5 hours. The pellet was dissolved in minimal volume of viral resuspension buffer (20-mM Tris pH 8.0, 250-mM NaCl, 10-mM $MgCl_2$, 5% sorbitol) and stored at -80°C until use. For introduction of PercevalHR RCAS into chicken embryos, fertile eggs were incubated for 32 hours such that embryos developed to HH8-9, followed by windowing of the egg and injection of concentrated virus into the coelomic cavity. Windowed eggs were resealed and incubated for an additional four days, after which time lungs were dissected and analyzed using confocal microscopy. PercevalHR images were acquired on a Nikon AXR scanning confocal microscope using a 20× 0.75 *NA* objective (CFI SuperFluor 20X MRF00200), imaging separately first with a 488-nm laser for λ_{high} and then with a 405-nm laser for λ_{low}, and collected with a 530/40 filter. The final ratiometric signal is a 32-bit division of $\lambda_{high}/\lambda_{low}$.

Image analysis

For fixed samples imaged at 10×, epithelial and mesenchymal regions were segmented in ImageJ by applying a threshold to the E-cadherin (Ecad) channel. Custom Julia code (available at github.com/bezlemma) was then used to remove the esophagus from the image. In cases where the esophageal signal was very close to that of the lung, Ecad signal was manually deleted in ImageJ. The original confocal volume data and associated segmentations are provided alongside the code.

Within these segmented regions, the mean Tom20 signal intensity was calculated separately for the epithelium and mesenchyme. To define proximal and medial sections, y-coordinates were used to identify the carina and the first branch; data between the carina and first branch were defined as the proximal region. Data halfway between the last visible branch and the distal tip were defined as the distal region. Data between these two regions were defined as the medial

bioRxiv preprint doi: https://doi.org/10.1101/2025.08.31.673364; this version posted August 31, 2025. The copyright holder for this preprint (which was not certified by peer review) is the author/funder, who has granted bioRxiv a license to display the preprint in perpetuity. It is made available under aCC-BY 4.0 International license.

region. Julia code for both segmentation and mean-intensity calculations (with y-coordinates) are included in the repository.

For both fixed and live samples imaged at 20×, manual measurements were performed to calculate the ratio of fluorescence intensities—Tom20 (mitochondrial density), MitoView633 (mitochondrial potential), or ATPRed1 (mitochondrial ATP)—between the dorsal and ventral epithelium. These measurements were made in four specified regions by using the ImageJ lasso selection tool.

In continuous temporal confocal datasets, the images were first stabilized in ImageJ. A square selection was placed over the epithelial area of interest at each time point, and the mean intensity within this selection was recorded. For continuous spatial data, a freehand line tool with a width corresponding to the approximate thickness of the epithelium (~30 µm) was used to measure mean intensity across that width. All datasets were then manually aligned so that branching epithelial regions overlapped. The raw data from these manual steps are included within the plotting scripts provided in the code repository.

Spatial transcriptomic mapping

We performed spatial transcriptomic mapping using a modified microfluidic-enabled, deterministic barcoding-based (DBiT-seq) workflow (*37*). Briefly, lungs from 124-hour-old embryos were embedded and sectioned into 7–9-µm-thick slices using a cryostat, then mounted at the center of poly-L-lysine-coated glass slides. The sections underwent spatial barcoding through two rounds of barcode application. In the first round, barcodes A1 to A50 were introduced into the tissue sections via 15-µm-wide microfluidic channels, followed by in situ reverse transcription. In the second round, barcodes B1 to B50 were applied orthogonally to the first set using another microfluidic device, accompanied by in situ ligation. The barcoded tissue sections were subsequently digested, and the resulting cDNA was purified and sequenced according to established protocols (*38*).

Oxygen consumption rate measurements

bioRxiv preprint doi: https://doi.org/10.1101/2025.08.31.673364; this version posted August 31, 2025. The copyright holder for this preprint (which was not certified by peer review) is the author/funder, who has granted bioRxiv a license to display the preprint in perpetuity. It is made available under aCC-BY 4.0 International license.

Lung explants were cultured at 37°C and 90% humidity in Agilent Seahorse XF DMEM, pH 7.4 (Agilent #103575-100), supplemented with 5% FBS, antibiotics (50 units/mL of penicillin and streptomycin), glucose (10 mM; Agilent #103577-100), pyruvate (1 mM; Agilent #103578-100), and glutamine (2 mM; Agilent #103579-100). Prior to culture, Islet Capture Microplates (Agilent #101122-100) were coated with a central circle of 2-μL poly-D-lysine. Freshly dissected lung explants were placed in the middle of the circle, submerged in 500-μL media, and then covered with nylon islet capture screens. Images of each explant were acquired using an Olympus SZX7 stereoscope fitted with an OMAX A35140U camera to permit normalization of the data by projected area of the tissue. Data were discarded from any wells in which lungs had been displaced out of the central circle during the process. Oxygen consumption rates were measured using an Agilent Seahorse XFe24 Analyzer following the manufacturer's protocols; pharmacological agents were injected at the concentrations indicated, with 56 μL in Port 1 and 62 μL in Port 2.

Reaction-diffusion model of ATP hydrolysis

We model the concentrations of [ATP] and [ADP] in a 1D domain [0, L] that represents the apicobasal axis of the cell. Each chemical species satisfies a diffusion equation with spatially dependent reaction terms

$$\partial_t[ATP] = D\partial_x^2[ATP] + R(x, [ATP], [ADP]),$$
$$\partial_t[ADP] = D\partial_x^2[ADP] - R(x, [ATP], [ADP]).$$

Using finite difference, we numerically calculate the density of chemical species A as

$$A(x, t + \Delta t) = A(x, t) + \Delta t\left[D\frac{A(x + \Delta x, t) - 2A(x, t) + A(x - \Delta x, t)}{(\Delta x)^2} \pm R(x, [ATP], [ADP])\right].$$

At the boundaries we impose a no-flux boundary condition:

$$\partial_x[A]|_{x=0,L} = 0.$$

This condition is implemented numerically by modifying the diffusion term at the boundaries to

$$\frac{2 * A(\Delta x, t) - A(0, t)}{(\Delta x)^2},$$

bioRxiv preprint doi: https://doi.org/10.1101/2025.08.31.673364; this version posted August 31, 2025. The copyright holder for this preprint (which was not certified by peer review) is the author/funder, who has granted bioRxiv a license to display the preprint in perpetuity. It is made available under aCC-BY 4.0 International license.

$$\frac{2 * A(L - \Delta x, t) - A(L, t)}{(\Delta x)^2}.$$

The reaction term R includes a baseline conversion of ATP to ADP everywhere, representing the cell's baseline ATP usage, R_0. The reaction term also includes an ADP-to-ATP conversion (phosphorylation), $R^{ADP \to ATP}$, which applies proportionally to a mitochondrial density function $M(x)$. Finally, the reaction term includes an ATP-to-ADP conversion term (hydrolysis), $R^{ATP \to ADP}$, which applies proportionally to actomyosin density $\Theta(x)$:

$$R(x, [ATP], [ADP]) = -R_0 + R^{ADP \to ATP}([ADP]) \times M(x) - R^{ATP \to ADP}([ATP]) \times \Theta(x).$$

The phosphorylation term is such that no timestep phosphorylates more ADP than is available.

$$R^{ADP \to ATP}([ADP]) = \min\left(R_0^{ADP \to ATP}, \frac{[ADP]}{\Delta t}\right).$$

The hydrolysis term has a Michaelis-Menten-like dependence, with [ADP] as an additional inhibitor with $K_i = K_m$, such that

$$R^{ATP \to ADP} = \min\left(R_{max}^{ATP \to ADP} \frac{[ATP]}{K_m + [ATP] + [ADP]}, \frac{[ATP]}{\Delta t}\right).$$

Where $R_{max}^{ATP \to ADP}$ is the maximum hydrolysis rate, K_m is the Michaelis constant, and a minimum function is used to ensure that enough ATP exists for the step to take place. $R_0^{ADP \to ATP}$ is calculated from experimental measurements of mitochondrial respiration. Simulations are shown with D=100 $\frac{\mu m^2}{s}$, $R_{max}^{ATP \to ADP} = 750 \frac{\mu M}{s} / \mu m$, K_m=[10:900], and $R_0 = 15 \frac{\mu M}{s} / \mu m$.

Estimation of ATP phosphorylation rate from experimental measurements of oxygen consumption rate

To estimate the rate of ATP production from mitochondria, $\partial_t [ATP]_{mito}$, from the total rate of oxygen consumption, $\partial_t [O_2]$, we measured the rate of oxygen consumption when the ATP-producing complex V of mitochondria was inhibited by treatment with oligomycin, $\partial_t [O_2]_{other}$. This term, $\partial_t [O_2]_{other}$, encompasses both the mitochondrial proton leak as well as oxygen consumption due to non-mitochondria-related events. Thus, the oxygen consumption rate due to mitochondria is

bioRxiv preprint doi: https://doi.org/10.1101/2025.08.31.673364; this version posted August 31, 2025. The copyright holder for this preprint (which was not certified by peer review) is the author/funder, who has granted bioRxiv a license to display the preprint in perpetuity. It is made available under aCC-BY 4.0 International license.

$$\partial_t[O_2]_{mito} \approx \partial_t[O_2] - \partial_t[O_2]_{other}. \quad \textbf{Eq. 1}$$

Since there are approximately 5.5 molecules of ATP produced per molecule of O_2 consumed (*39*), we calculate mitochondrial ATP production as

$$\partial_t[ATP]_{mito} \approx 5.5(\partial_t[O_2]_{mito}) \approx 5.5(\partial_t[O_2] - \partial_t[O_2]_{other}) \, . \quad \textbf{Eq. 2}$$

The energy produced by phosphorylating ADP into ATP, $E_{ADP\to ATP}$, has both a constant term, $E^0_{ATP\to ADP}$, and an entropic term dependent on the logarithm of the ADP/ATP ratio. Specifically,

$$E_{ADP\to ATP} = E^0_{ATP\to ADP} + \ln\left(\frac{[ADP][P_i]}{[ATP][H_2O]}\right). \quad \textbf{Eq. 3}$$

To estimate the chemical energy produced by mitochondria from oxygen consumption rates, we take the ADP/ATP ratio such that $E_{ADP\to ATP} \approx 10^{-19}J$ uniformly (*27*). We convert this energy into power, or energy per unit time:

$$\partial_t E_{mito} = \partial_t[ATP]_{mito} \times E_{ADP\to ATP} \, . \quad \textbf{Eq. 4}$$

The resulting estimated chemical power of ATP phosphorylation produced by mitochondria, $\partial_t[ATP]_{mito}$, is $\sim 60\ \mu J/min$ or $\sim 1 \mu W$. Over several hours this accounts for a chemical energy that is 100-million-fold the mechanical deformation energy derived below.

Calculation of viscoelastic energy requirements for the initiation of a branch by apical constriction

Viscoelastic energy change from change in volume, $\Delta E_{stretch}$, has an elastic energy density term plus a viscous dissipation term:

$$\Delta E_{stretch}/V = \frac{1}{2}K\epsilon^2 + \frac{1}{2}\int \mu\dot{\epsilon}^2 dt \, .$$

Where K is the bulk modulus, ϵ is the volumetric strain, and μ is the viscosity. Over time, Δt, this becomes

bioRxiv preprint doi: https://doi.org/10.1101/2025.08.31.673364; this version posted August 31, 2025. The copyright holder for this preprint (which was not certified by peer review) is the author/funder, who has granted bioRxiv a license to display the preprint in perpetuity. It is made available under aCC-BY 4.0 International license.

$$\Delta E_{stretch}/V = \frac{1}{2}K\epsilon^2 \;\; + \frac{1}{2}\mu(\frac{\Delta\epsilon}{\Delta t})^2\Delta t \, .$$

Consider the deformation of a half-sphere with radius R_1 from a flat sheet as a proxy for the deformations studied here:

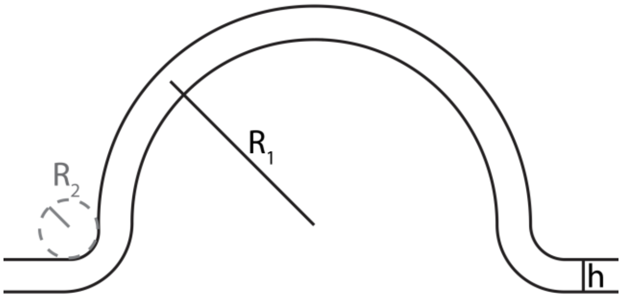

Note that the volumetric strain, $\epsilon = \frac{\Delta V}{V_0} = \frac{V_f - V_0}{V_0}$, is $\epsilon = 1$, as a circular patch with height h and radius R_1 gives $V_0 = h\pi R_1^2$, while deformation into a half sphere gives $V_f = h2\pi R_1^2$. Thus,

$$\Delta E_{stretch}/V = \frac{1}{2}(K + \frac{\mu}{\Delta t}) \; .$$

Taking physical constants as $K \approx 10\ kPa$, $\Delta t \approx 4\ hrs$, $\mu \approx 10\ Pa \cdot s$, $h = 30\ \mu m$, and $R_1 = 100\ \mu m$, we find that $\Delta E_{stretch} \approx 5\ nJ$.

To calculate the change in energy due to bending, we assume a form of local bending energy density with principal curvatures κ_1 and κ_2 to be

$$u_b = \frac{1}{2}D[(\kappa_1 + \kappa_2)^2 - 2(1-\nu)\kappa_1\kappa_2],$$

where $D = \frac{Eh^3}{12(1-\nu^2)}$ is the flexural rigidity. Taking this for our sphere, where $\kappa_1 = \kappa_2 = \frac{1}{R_1}$, we integrate the local bending energy density of a thin sheet with curvature R_1,

$$u_{b,sphere} = \frac{Eh^3}{12(1-\nu^2)}\frac{1+\nu}{R_1^2},$$

over the area of the formed half sphere

$$\Delta E_{bend,sphere} = \int \frac{Eh^3}{12(1-\nu^2)}\frac{1+\nu}{R_1^2}dA = \frac{\pi E h^3}{6(1-\nu^2)}(1+\nu).$$

bioRxiv preprint doi: https://doi.org/10.1101/2025.08.31.673364; this version posted August 31, 2025. The copyright holder for this preprint (which was not certified by peer review) is the author/funder, who has granted bioRxiv a license to display the preprint in perpetuity. It is made available under aCC-BY 4.0 International license.

Then considering the neck region, where $\kappa_1 = -\frac{1}{R_2}, \kappa_2 = \frac{1}{R_1} \ll |\kappa_1|$, the local bending energy density is approximately

$$u_{b,sphere} = \frac{Eh^3}{24(1-\nu^2)}\frac{1}{R_2^2}.$$

By integrating over the neck region with area $\approx 2\pi R_1 R_2$ we obtain the bending energy of the neck as

$$\Delta E_{bend,neck} \approx \frac{\pi E h^3}{12(1-\nu^2)}\frac{R_1}{R_2}.$$

The total bending energy is thus

$$\Delta E_{bend} = \Delta E_{bend,sphere} + \Delta E_{bend,neck} \approx \frac{\pi E h^3}{12(1-\nu^2)}(R_1/R_2 + 2(1+\nu)).$$

with radius $R_1 \approx 100\ \mu m$, and a curvature of $R_2 \approx 5\ \mu m$, Young's modulus $E \approx 10\ kPa$, thickness of the epithelium $h \approx 30\ \mu$m, and Poisson ratio $\nu \approx 0.3$. These parameters result in the bending energy $\Delta E_{bend} \approx 2\ nJ$.

Thus, to an order of magnitude, the total energy due to this idealized deformation is

$$\Delta E_{bend} + \Delta E_{stretch} \approx 10\ nJ\ .$$

Computational model

The 3D simulations of epithelial morphogenesis in response to apical constriction use a compressible Neo-Hookean hyperelastic constitutive law, solved via the finite element method (FEM). We assumed that actomyosin-driven apical constriction is much slower than mechanical relaxation and, hence, the system is always in quasi-mechanical equilibrium, which was achieved by minimizing the total elastic energy as described below.

Supposing that the initial reference volume is Ω, we introduced a fixed Cartesian coordinate system with an orthonormal basis $\{\boldsymbol{e_1}, \boldsymbol{e_2}, \boldsymbol{e_3}\}$ and spatial coordinates given as (X_1, X_2, X_3), denoted as $\boldsymbol{X} = X_i \boldsymbol{e_i}$ where summation over repeated indices is implied. At some later time t, the

bioRxiv preprint doi: https://doi.org/10.1101/2025.08.31.673364; this version posted August 31, 2025. The copyright holder for this preprint (which was not certified by peer review) is the author/funder, who has granted bioRxiv a license to display the preprint in perpetuity. It is made available under aCC-BY 4.0 International license.

system was deformed to a volume Ω_t and the Cartesian coordinates were mapped to a different vector field, denoted as $\boldsymbol{x} = \varphi_t(\boldsymbol{X})$. Using this notation, we follow finite deformation theory (*40*) and define the deformation gradient tensor as $F_{ij} = \frac{\partial x_i}{\partial X_j}$.

To implement apical constriction, we decompose the total deformation gradient $\boldsymbol{F}(\boldsymbol{X})$ as $\boldsymbol{F} = \boldsymbol{F_e}\boldsymbol{F_c}$, where it is assumed that isotropic contractions induce an intermediate stress-free state with deformation gradient $\boldsymbol{F_c} = (1 - t\beta)\boldsymbol{I}$, where $\beta(\boldsymbol{X})$ is the contractility field and t is time, and that there is an additional elastic deformation of this intermediate stress-free state to the final deformed state due to the elastic deformation gradient $\boldsymbol{F_e}$. The total deformation gradient can be expressed in terms of the displacement field $\boldsymbol{u}(\boldsymbol{X}) = \boldsymbol{x}(\boldsymbol{X}) - \boldsymbol{X}$ as $\boldsymbol{F} = \boldsymbol{I} + \nabla\boldsymbol{u}$. Thus, the elastic deformation gradient can be expressed as $\boldsymbol{F_e} = \boldsymbol{F}\boldsymbol{F_c}^{-1} = (1 - t\beta)^{-1}(\boldsymbol{I} + \nabla\boldsymbol{u})$.

To account for large material deformation, we assumed the tissues to be Neo-Hookean solids with the elastic energy storage density $\psi(\boldsymbol{F_e}, \boldsymbol{X})$ defined as

$$\psi = \frac{\lambda}{2}\ln(J)^2 + \frac{\mu}{2}(tr(\boldsymbol{C}) - 3 - 2\ln(J)).$$

In which $\lambda(\boldsymbol{X})$ and $\mu(\boldsymbol{X})$ are the Lamé constants, $\boldsymbol{C}$ is the Cauchy-Green deformation tensor, and J is the Jacobian of the elastic deformation gradient $\boldsymbol{F_e}$. Explicitly,

$$\lambda = \frac{E}{2(1+\nu)},$$

$$\mu = \frac{E\nu}{(1-2\nu)(1+\nu)},$$

$$\boldsymbol{C} = \boldsymbol{F_e}^T\boldsymbol{F_e},$$

$$J = \det(\boldsymbol{F_e}),$$

where ν is the Poisson ratio and E is the elastic modulus.

The FEM, a standard method in computational mechanics to numerically solve for the deformation field under given boundary conditions (*41*), was used to calculate the displacement field $\boldsymbol{u}(\boldsymbol{X})$ that minimizes the total potential energy $\Pi(\boldsymbol{u}, \boldsymbol{\lambda}_{tr}, \boldsymbol{\lambda}_{rot})$ for a prescribed contraction

bioRxiv preprint doi: https://doi.org/10.1101/2025.08.31.673364; this version posted August 31, 2025. The copyright holder for this preprint (which was not certified by peer review) is the author/funder, who has granted bioRxiv a license to display the preprint in perpetuity. It is made available under aCC-BY 4.0 International license.

profile where the Lagrange multipliers $\boldsymbol{\lambda}_{tr}$and $\boldsymbol{\lambda}_{rot}$ constrain rigid body translation and rotation, respectively. The total potential energy can be written as (*42*):

$$\Pi(\boldsymbol{u}, \boldsymbol{\lambda}_{tr}, \boldsymbol{\lambda}_{rot}) = \int_{\Omega} \psi(\boldsymbol{F_e}, \boldsymbol{X})\mathrm{d}V - \int_{\Omega} \boldsymbol{\lambda}_{tr} \cdot \boldsymbol{u}\, dV - \int_{\Omega} \boldsymbol{\lambda}_{rot} \cdot (\boldsymbol{X} \times \boldsymbol{u})\, dV.$$

In all simulations we use traction-free boundaries. For the neural tube geometry (**Fig. S5b,d**), a boundary condition enforces that the z-displacement at top-z of the geometry is 0, preventing the planar parts of the geometry from folding upwards. The unknown displacement field $\boldsymbol{u}$ is then obtained via the variation of the potential energy function $\Pi(\boldsymbol{u}, \boldsymbol{\lambda}_{tr}, \boldsymbol{\lambda}_{rot})$ with respect to $\boldsymbol{u}$, $\boldsymbol{\lambda}_{tr}$, and $\boldsymbol{\lambda}_{rot}$ and solving,

$$\delta\Pi = \int_{\Omega} \frac{\partial\psi(\boldsymbol{F_e}, \boldsymbol{X})}{\partial \boldsymbol{u}} \cdot \delta\boldsymbol{u}\, \mathrm{d}V - \int_{\Omega} \boldsymbol{\lambda}_{tr} \cdot \delta\boldsymbol{u}\, dV - \int_{\Omega} \boldsymbol{\lambda}_{rot} \cdot (\boldsymbol{X} \times \delta\boldsymbol{u})\, dV$$

$$- \int_{\Omega} \delta\boldsymbol{\lambda}_{tr} \cdot \boldsymbol{u}\, dV - \int_{\Omega} \delta\boldsymbol{\lambda}_{rot} \cdot (\boldsymbol{X} \times \boldsymbol{u})\, dV = 0.$$

For each timestep, we solve displacements $\boldsymbol{u}(\boldsymbol{X})$ by minimizing the total elastic energy Π using the Newton-Raphson method, then the next timestep occurs with an increase in contraction according to $\beta(\boldsymbol{X})$ and the process is repeated. If the Newton-Raphson method fails to converge, we reduce the timestep and repeat.

To numerically solve the above energy minimization problem, the domain Ω was discretized using first-order tetrahedral elements that were generated with the help of an open-source software Gmsh (*43*). Then, the potential energy minimization problem was implemented in the open-source computing platform FEniCS (*44*). Simulations contained ~20,000 tetrahedral elements, chosen by serial refinement until simulation results remained consistent. Geometrical parameters were chosen to match confocal imaging data. The non-dimensional elastic modulus was defined as $E = 1$ and the Poisson ratio was set as ν=0.3. All simulation code and geometries can be found at github.com/bezlemma. Simulations were visualized in ParaView (*45*).

bioRxiv preprint doi: https://doi.org/10.1101/2025.08.31.673364; this version posted August 31, 2025. The copyright holder for this preprint (which was not certified by peer review) is the author/funder, who has granted bioRxiv a license to display the preprint in perpetuity. It is made available under aCC-BY 4.0 International license.

Supplementary Figures

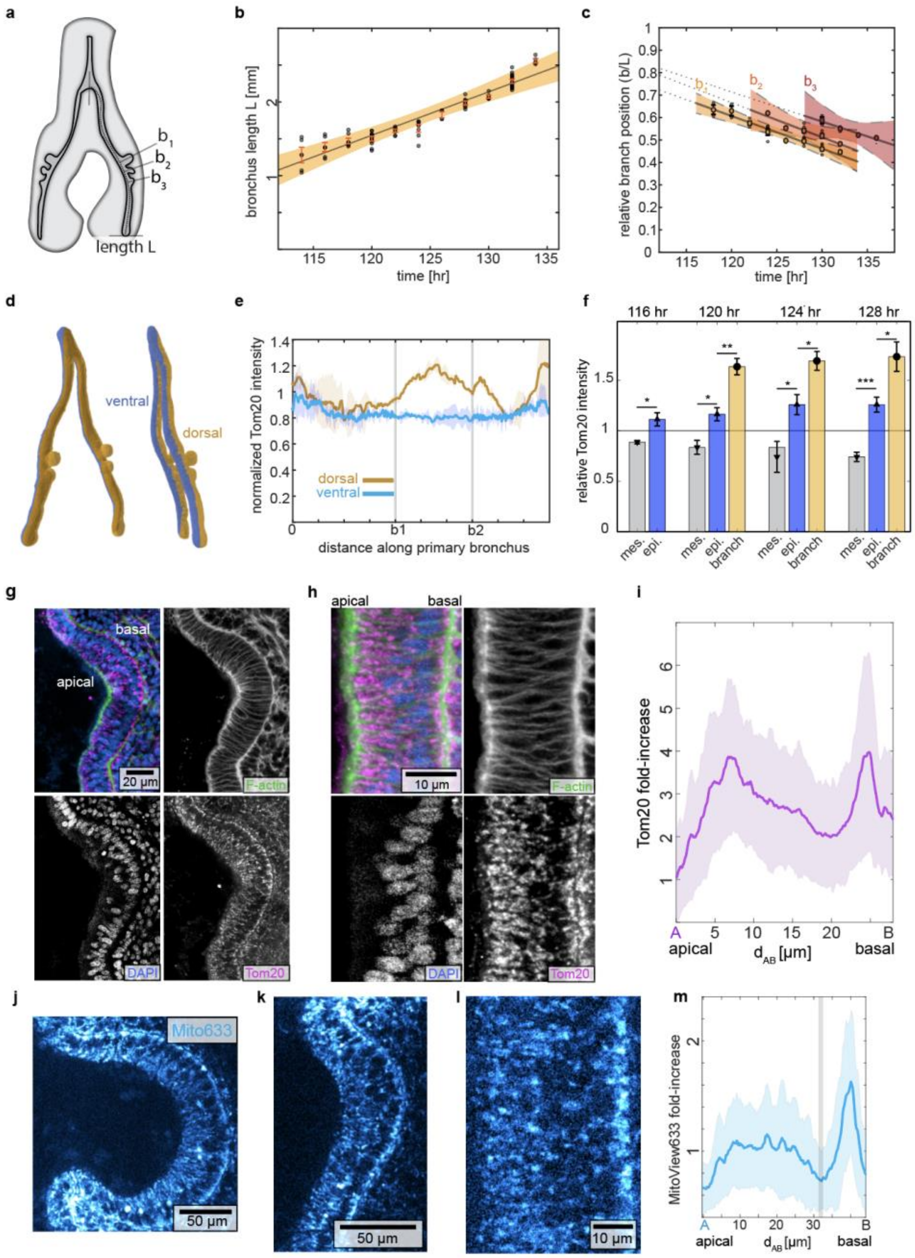

Fig. S1. Characterization of mitochondrial membrane density and potential in the early embryonic chicken lung – related to Fig. 1. (**a**) Schematic of HH26-stage lung, showing first branch (b1), second branch (b2), third branch (b3), and length of the primary bronchus (L). (**b**) Graph showing L as a function of developmental time. Each smaller dot represents one lung. Shaded region represents the 95% confidence interval of the linear fit. (**c**) Graph showing the relative positions of b1, b2, and b3 as a function of developmental time, normalized to L. Shown are data from 6 lungs for each timepoint. Shaded region represents the 95% confidence interval of the linear fits. (**d**) Volume rendering of epithelium within HH26-stage lung, color-coded to indicate dorsal (gold) and ventral (blue) regions of the epithelium. (**e**)

bioRxiv preprint doi: https://doi.org/10.1101/2025.08.31.673364; this version posted August 31, 2025. The copyright holder for this preprint (which was not certified by peer review) is the author/funder, who has granted bioRxiv a license to display the preprint in perpetuity. It is made available under aCC-BY 4.0 International license.

Graph of normalized Tom20 staining intensity in the dorsal (gold) and ventral (blue) epithelium, as a function of distance along the primary bronchus, normalized such that 1 is the mean Tom20 intensity of the lung. (**f**) Graph of Tom20 staining intensity in the mesenchyme, total epithelium, and branching epithelium as a function of developmental stage. Error bars indicate s.e.m. for 3 lungs at the 0-branch stage (116 hr) and 5 lungs at each subsequent stage. (*) indicates $p<0.051$, (**) $p<0.01$, (***) $p<0.001$, (**g**) Fluorescence images of F-actin (green), Tom20 (magenta), and nuclei (blue) in a single branch of a developing lung. Scale bar, 20 μm. (**h**) Fluorescence images of staining for F-actin (green), Tom20 (magenta), and nuclei (blue) at an uncurved future branch site in the dorsal epithelium. Scale bar, 10 μm. (**i**) Graph showing quantification of normalized Tom20 intensity as a function of position along the apicobasal axis, averaged along the proximal-distal axis of a dorsal branching region. Shaded error bar indicates standard deviation along the proximal-distal axis. Fluorescence images of MitoView633 dye visualizing mitochondrial membrane potential in (**j**) a branch, (**k**) a nascent branch, and (**l**) an uncurved future branch site in the dorsal epithelium. Scale bars, 50, 50, 10 μm. (**m**) Graph showing quantification of normalized MitoView633 intensity along the apicobasal axis of a branching region. Shaded error bar indicates standard deviation of integrated intensity.

bioRxiv preprint doi: https://doi.org/10.1101/2025.08.31.673364; this version posted August 31, 2025. The copyright holder for this preprint (which was not certified by peer review) is the author/funder, who has granted bioRxiv a license to display the preprint in perpetuity. It is made available under aCC-BY 4.0 International license.

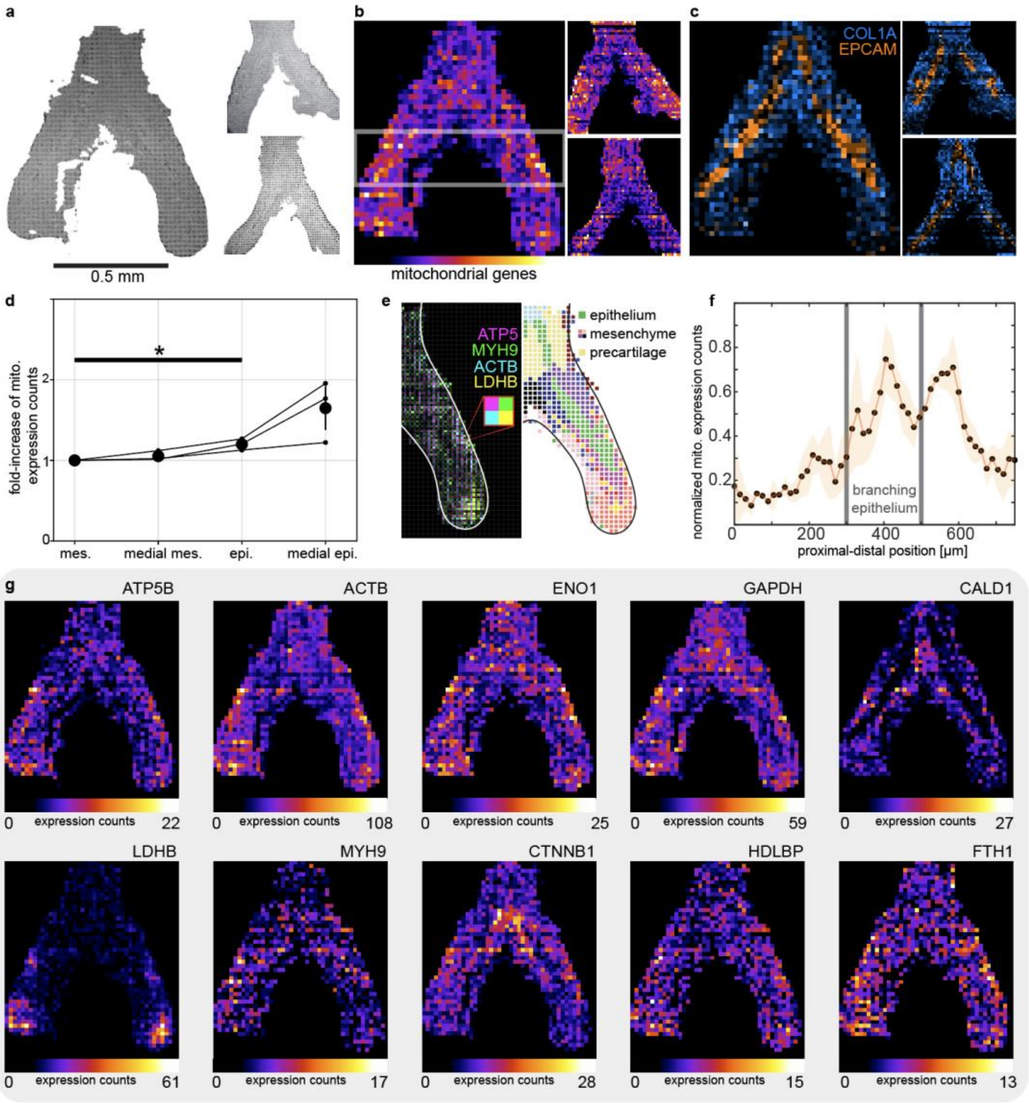

Fig. S2. Unbiased spatial transcriptomics reveals increased expression of mitochondrial genes in the branching lung epithelium – related to Fig. 1. (**a**) Segmented images of the three lung slices used for DBiTseq. (**b**) Merged expression counts of mitochondrial genes; white bars bracket the area considered as medial for later analysis. (**c**) Maps of COL1A and EPCAM expression used to segment the mesenchyme and epithelium, respectively. (**d**) Graph indicating fold increase of mitochondrial gene counts, as compared to the mesenchyme. Data show significant difference (p=0.03) in mitochondrial gene expression between the mesenchyme and epithelium. (**e**) Composite map of genes encoding for ATP synthase (ATP5), non-muscle myosin heavy chain (MYH9), beta-actin (ACTB), and lactate dehydrogenase b (LDHB). (**f**) Graph showing intensity of mitochondrial gene expression along the proximal-distal axis of the dorsal epithelium, defined as the outer third of the tissue, from carina to distal tip, revealing higher mitochondrial gene-expression counts in the branching and future branching regions of the lung. Shaded region is s.e.m. across three independent replicates. (**g**) Spatial maps of expression counts of ATP synthase F1β subunit (ATP5B), ACTB, enolase-1 (ENO1), glyceraldehyde 3-phosphate dehydrogenase (GAPDH), caldesmon (CALD1), LDHB, MYH9, beta-catenin (CTNNB1), high-density lipoprotein binding protein (HDLBP), and ferritin heavy chain (FTH1).

bioRxiv preprint doi: https://doi.org/10.1101/2025.08.31.673364; this version posted August 31, 2025. The copyright holder for this preprint (which was not certified by peer review) is the author/funder, who has granted bioRxiv a license to display the preprint in perpetuity. It is made available under aCC-BY 4.0 International license.

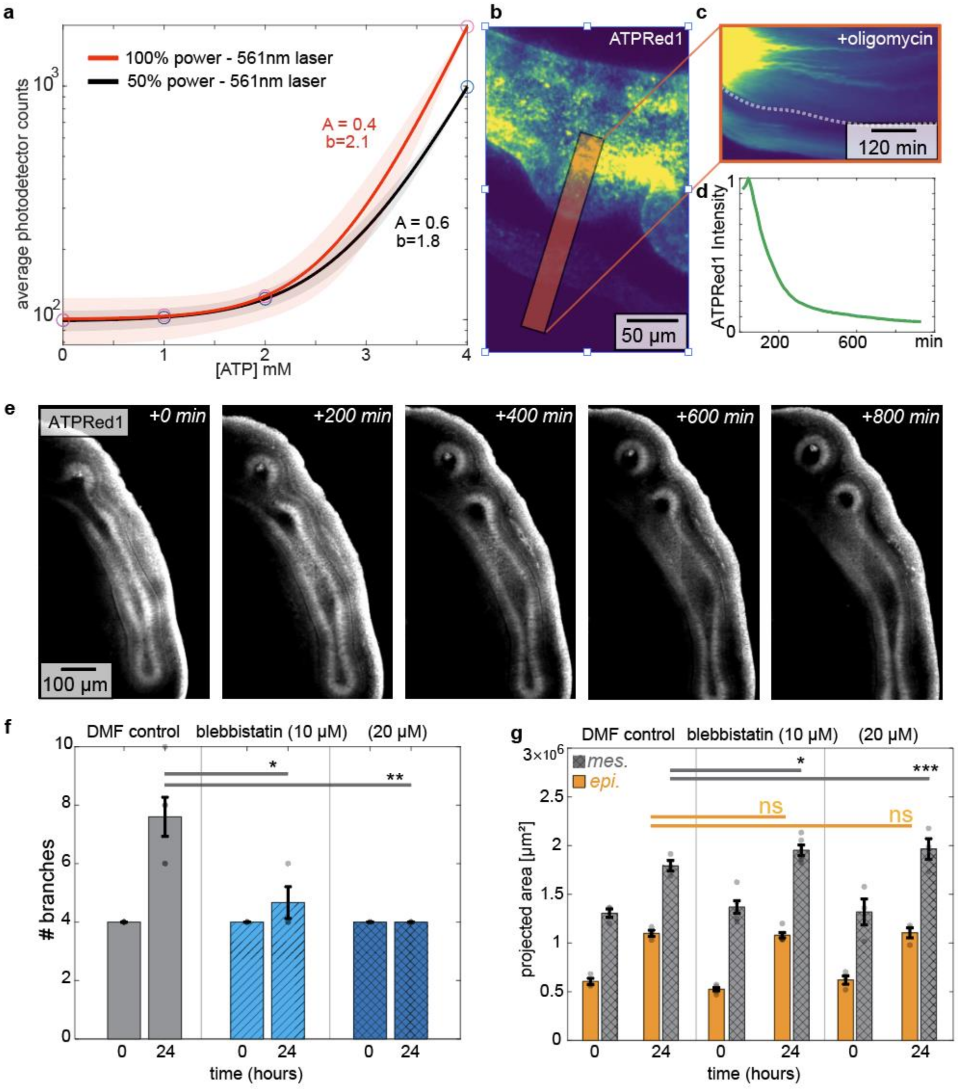

Fig. S3. Characterization of mitochondrial ATP and actomyosin contractility in the developing lung – related to Fig. 2. (**a**) Graph showing calibration of ATPRed1 fluorescence intensity as a function of ATP concentration [ATP] for two different laser intensities. Lines show the best exponential fits for the data. Shaded error bar indicates the 95% confidence interval of the fit. (**b**) Fluorescence image of ATPRed1 labeling of lung explant. Scale bar, 50 µm. (**c**) Kymograph of ATPRed1 fluorescence intensity as a function of time after treatment with oligomycin (2 µM), for region indicated in (b). Time bar, 120 min. (**d**) Graph showing change in relative ATPRed1 fluorescence intensity as a function of time after treatment with oligomycin. (**e**) Timelapse fluorescence images of z-projected average ATPRed1 intensity in embryonic lung explant. A single z-slice is shown in Movie S5. Scale bar, 100 µm. (**f**) Graph showing number of branches as a function of time in lung explants cultured in the presence of DMF control or blebbistatin (10 µM or 20 µM). Error bars indicate s.e.m. for 3 explants across 3 independent experiments. *, ** indicate p=0.04, p=0.004. (**g**) Graph showing projected area of the epithelium and mesenchyme as a function of time in lung explants cultured in the presence of DMF control or blebbistatin (10 µM or 20 µM). Error bars indicate s.e.m. for 6 explants across 3 independent experiments. *, ** indicate p=0.04, p=0.001.

bioRxiv preprint doi: https://doi.org/10.1101/2025.08.31.673364; this version posted August 31, 2025. The copyright holder for this preprint (which was not certified by peer review) is the author/funder, who has granted bioRxiv a license to display the preprint in perpetuity. It is made available under aCC-BY 4.0 International license.

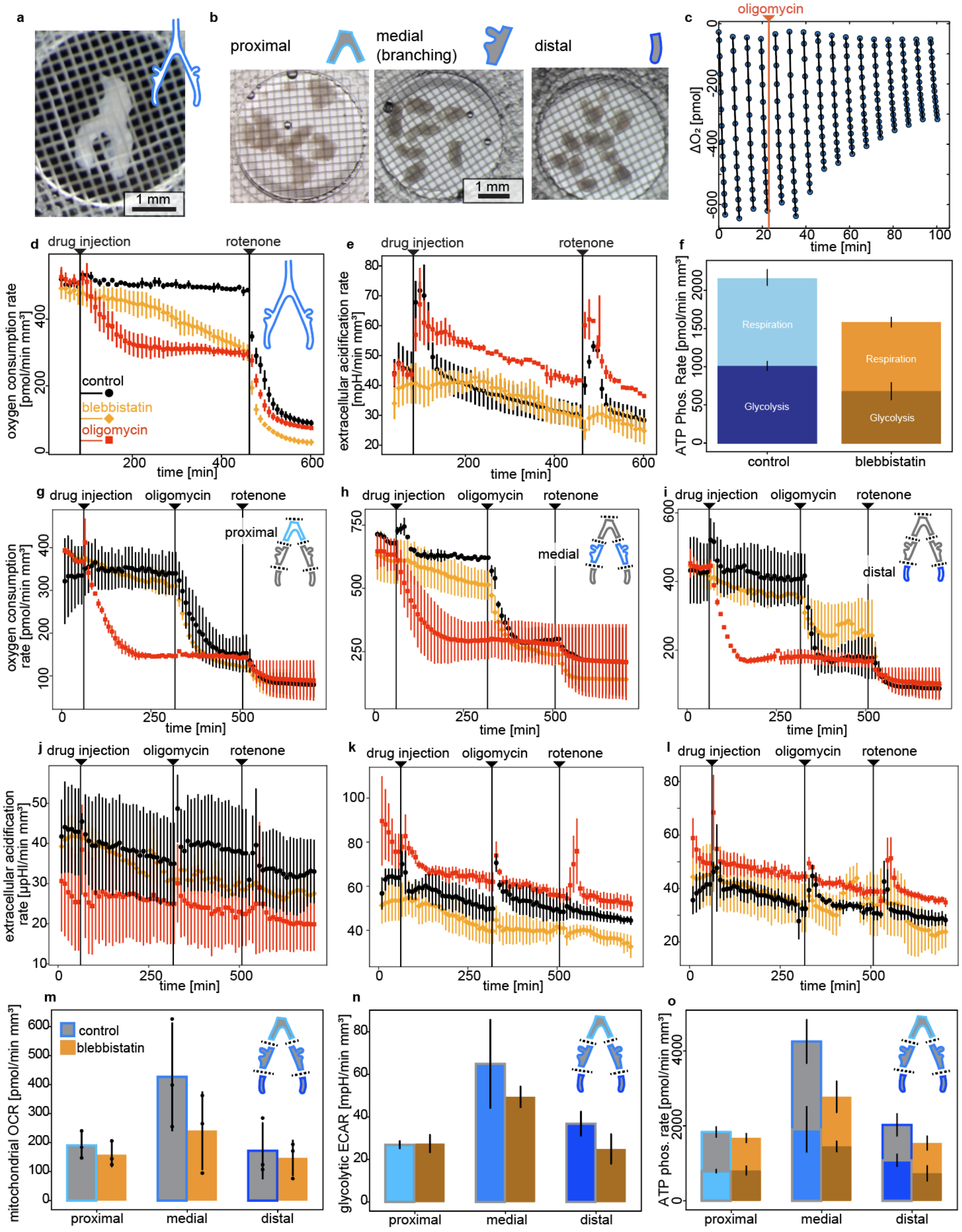

Fig. S4. Characterization of oxygen consumption rate and extracellular acidification rate in embryonic lung explants – related to Fig. 2. (**a**) Brightfield image of an intact lung placed in a Seahorse XFe24 Islet Well with a containing grid on top of it. Scale bar, 1 mm. (**b**) Brightfield images of proximal, medial, and distal portions of lungs pooled in XFe24 Islet Wells. Scale bar, 1 mm. (**c**) Graph showing an example raw measurement of oxygen levels. Between each line, vibrations reoxygenate the media in the well before the next series of measurements is taken. Dots represent oxygen readings, while the slopes of the line fits are the oxygen consumption rates. (**d**) Graph showing oxygen consumption rates for intact

bioRxiv preprint doi: https://doi.org/10.1101/2025.08.31.673364; this version posted August 31, 2025. The copyright holder for this preprint (which was not certified by peer review) is the author/funder, who has granted bioRxiv a license to display the preprint in perpetuity. It is made available under aCC-BY 4.0 International license.

lungs treated with oligomycin, blebbistatin, or DMF control, followed by a rotenone injection. Error bars are the standard error over 3 experimental replicates; each replicate contains 16 lungs each measured in a separate well. (**e**) Graph showing extracellular acidification rates for the same data sets as in (d). (**f**) Graph showing calculated ATP phosphorylation rate due to mitochondrial respiration and glycolysis for intact lungs and lungs exposed to blebbistatin. Error bars are standard error over the experimental replicates. (**g-i**) Graphs showing oxygen consumption rates for pools of proximal, medial, and distal portions of 24 lungs. Error bars indicate standard error from 3 experimental replicates. (**j-l**) Graphs showing extracellular acidification rates for the same data sets as in (g-i). (**m**) Graph showing mitochondrial oxygen consumption rates calculated from the difference between the control/blebbistatin measurements and the oligomycin measurements. (**n**) Graph showing glycolytic extracellular acidification rates calculated from the value of control/blebbistatin rates after rotenone injection. (**o**) Graph showing the extrapolated ATP phosphorylation rate due to mitochondrial respiration and glycolysis for intact lungs and lungs exposed to blebbistatin. Error bars represent s.e.m.

bioRxiv preprint doi: https://doi.org/10.1101/2025.08.31.673364; this version posted August 31, 2025. The copyright holder for this preprint (which was not certified by peer review) is the author/funder, who has granted bioRxiv a license to display the preprint in perpetuity. It is made available under aCC-BY 4.0 International license.

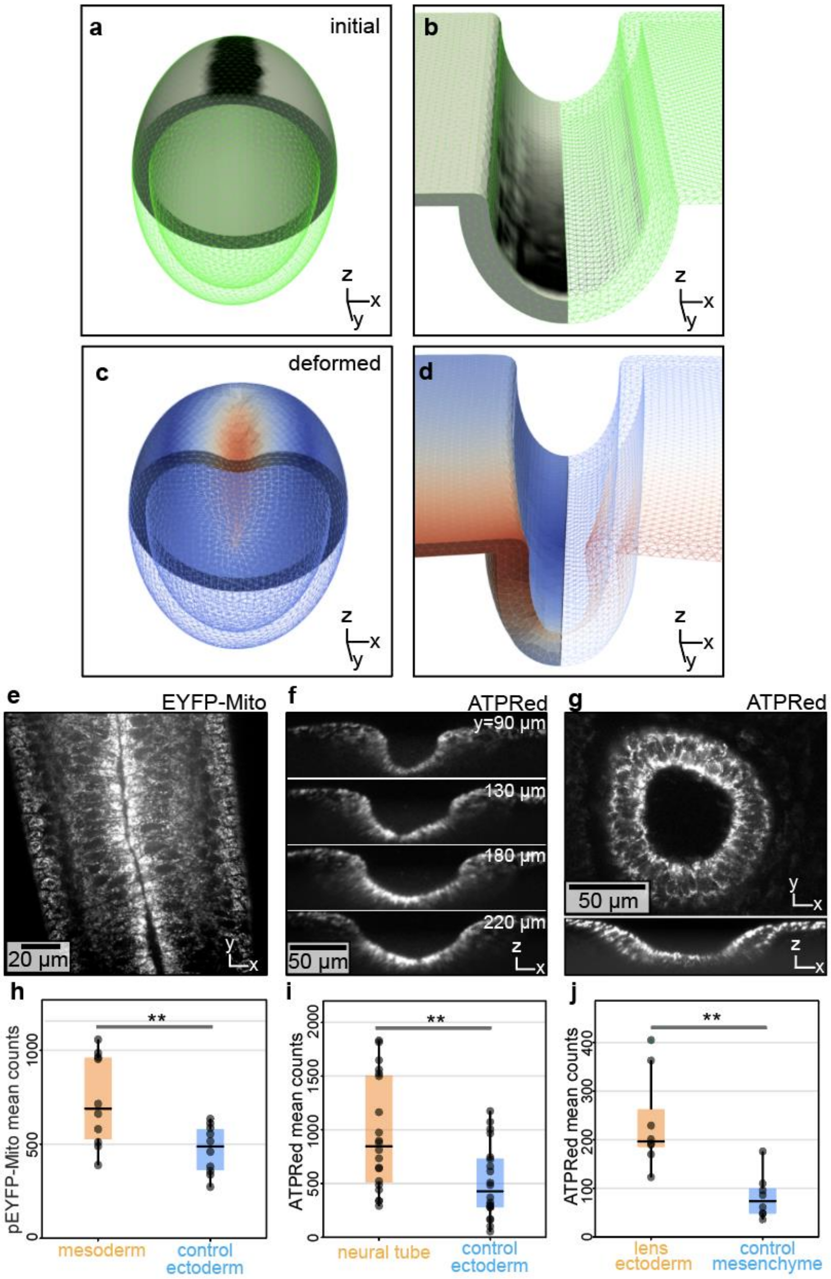

Fig. S5. Active strain simulations of apical constriction across species and tissues correspond to apicobasal patterns of enriched mitochondria – related to Fig. 4. (**a-b**) Cutaway view of initial geometries for simulations based on mitochondrial patterning of deformations during formation of the ventral furrow and neural tube; numerical mesh drawn in green, with applied active stress field shown in grayscale. (**c-d**) Cutaway view of numerical meshes deformed by active stresses from (a-b), colored by the displacement field. Live imaging of (**e**) pEYFP-mito intensity in *Drosophila* ventral furrow and ATPRed1 in (**f**) chick neural tube or (**g**) mouse lens placode. (**h-j**) Graphs showing mean intensity of pEYFP-mito intensity (p=0.0098) or ATPRed1 (p=0.0035, p=0.0027) as a function of position in each tissue.

bioRxiv preprint doi: https://doi.org/10.1101/2025.08.31.673364; this version posted August 31, 2025. The copyright holder for this preprint (which was not certified by peer review) is the author/funder, who has granted bioRxiv a license to display the preprint in perpetuity. It is made available under aCC-BY 4.0 International license.

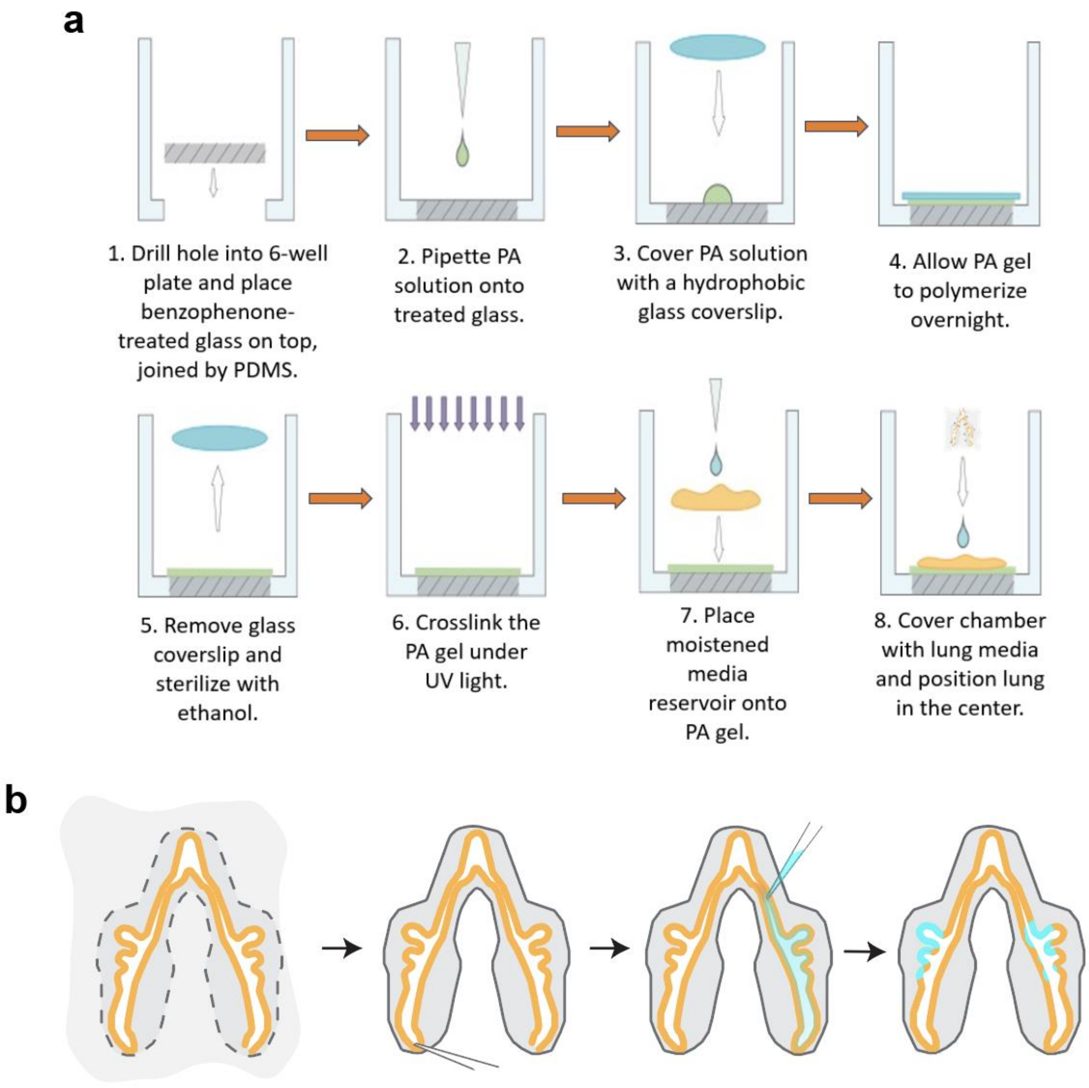

Fig. S6. Schematics of live-imaging setup. (**a**) Schematic illustrating construction of chambers for live imaging lungs at high NA. (**b**) Steps for injecting dye into the lumen of the chicken lung. First, the embryonic lung is resected from the embryo. Second, a pulled-glass pipette is used to make small openings at the distal tips to facilitate fluid flow. Third, dye is introduced near the trachea. Finally, the dye passes from the lumen into the epithelial cells.
